# Health care utilization for premature ventricular complexes in acute settings

**DOI:** 10.1016/j.hroo.2026.04.026

**Published:** 2026-05-08

**Authors:** Jean Jacques Noubiap, Gabrielle C. Montenegro, Janet J. Tang, Thomas A. Dewland, Edward P. Gerstenfeld, Gregory M. Marcus

**Affiliations:** Division of Cardiology, Department of Medicine, University of California-San Francisco, San Francisco, California

**Keywords:** Premature ventricular complexes, Arrhythmia, Prevalence, Catheter ablation, California, United States

## Abstract

**Background:**

Premature ventricular complexes (PVCs) are common; however, their burden in acute health care settings is not well characterized. In parallel, therapies, particularly catheter ablation, have evolved, but contemporary trends in utilization remain unclear.

**Objective:**

To evaluate the trends in health care utilization for PVCs.

**Methods:**

We conducted an analysis of California statewide health care data from 2016 to 2022, including adults aged ≥18 years seen in an ambulatory surgery unit, emergency department, or inpatient hospital. PVCs and comorbidities were identified using International Classification of Diseases, Tenth Revision, Clinical Modification (ICD-10-CM) codes as primary or secondary diagnoses, and catheter ablation using ICD-10-Procedure Coding System and Current Procedural Terminology codes.

**Results:**

Among 87,819,413 health care encounters from 20,280,466 adults, 235,848 (0.27%) included a PVC diagnosis and 22,806 (0.03%) had PVCs as a primary diagnosis. Patients with PVCs were older, more often male, and white, and had more cardiovascular comorbidities. The proportion of encounters with PVCs increased from 0.19% in 2016 to 0.31% in 2022. Encounters with PVCs as the primary diagnosis rose from 0.023% to 0.029%. Between 2016 and 2022, 3394 catheter ablations for PVCs were performed, nearly doubling from 343 in 2016 to 619 in 2022. Of the 21,265 patients who had at least 1 principal diagnosis of PVCs, 3278 (15.4%) underwent catheter ablation for PVCs. Ablation recipients were older, predominantly male, and white, and had greater cardiovascular comorbidity.

**Conclusion:**

PVCs, though infrequently coded as a primary diagnosis, are increasingly recognized in acute health care settings. The doubling of ablation procedures underscores growing clinical attention and the expanding role of interventional management, particularly among high-risk patients.

## Graphical abstract

**Figure:**
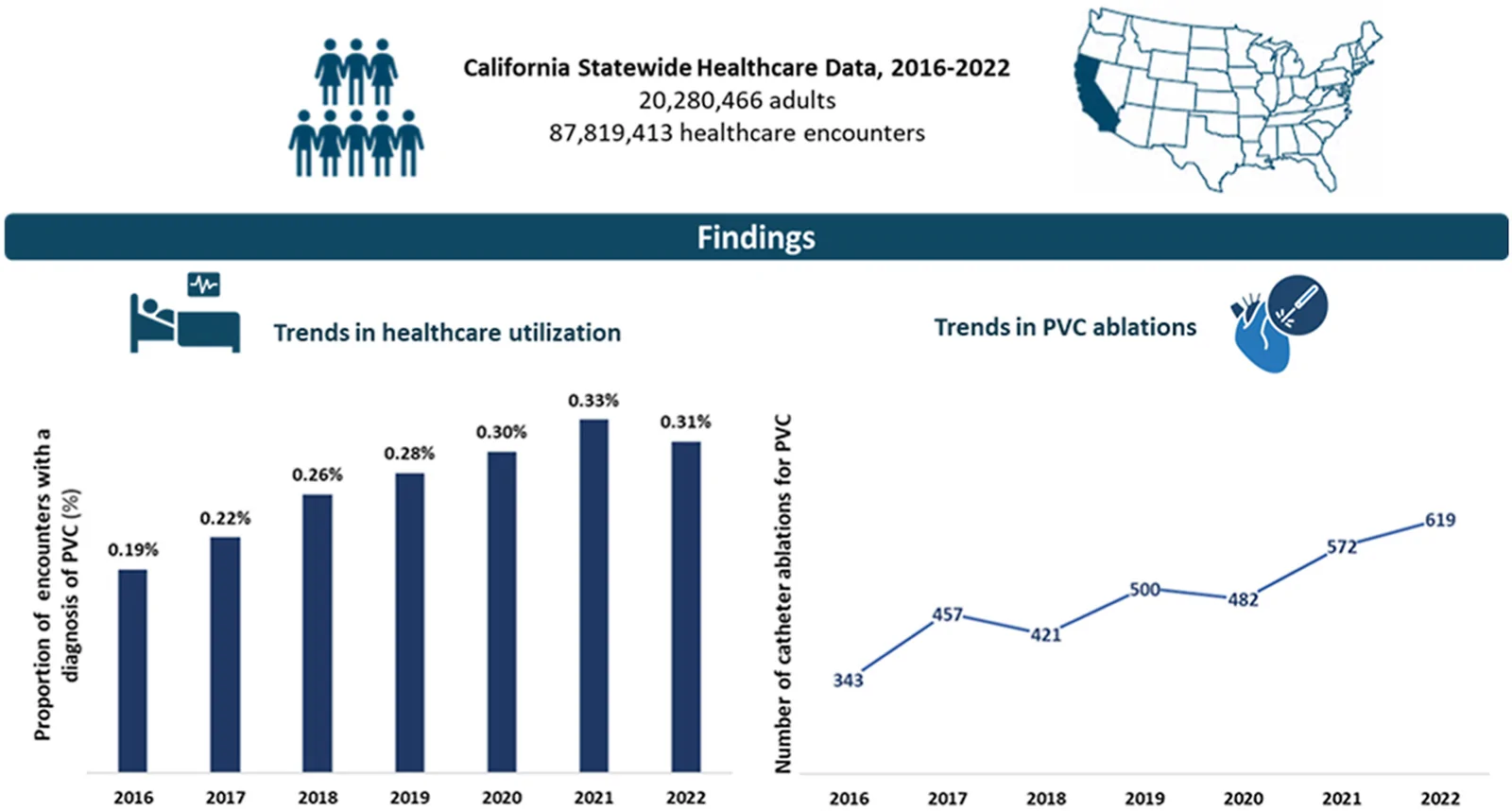

**Key Findings**

▪ Among over 87.8 million healthcare encounters in California (2016–2022), 0.27% were associated with a diagnosis of premature ventricular contractions (PVCs), and 0.03% were primarily for PVCs.
▪ The proportion of encounters with a diagnosis of PVCs increased over time, from 0.19% in 2016 to 0.31% in 2022, with higher rates in men, older individuals, and white patients.
▪ Health care utilization specifically for PVCs (primary diagnosis) also increased significantly over the study period across all sociodemographic groups.
▪ The number of catheter ablations for PVCs nearly doubled between 2016 and 2022, with approximately 15% of patients undergoing ablation.

## Introduction

Premature ventricular complexes (PVCs) are very common and frequently identified incidentally on electrocardiography or cardiac monitoring.^1^ The prevalence of PVCs increases with age and co-existing cardiovascular risk factors and disease.^1^^,^^2^ In 1 population-based study, 69% of young healthy individuals demonstrated PVCs on 24-hour Holter monitoring.^2^ In the Multi-Ethnic Study of Atherosclerosis (MESA) study, 99.5% of elderly participants had at least 1 PVC over a median of 14 days of monitoring.^3^ Importantly, with the increasing availability and use of direct-to-consumer wearables with electrocardiogram (ECG) capabilities such as the Apple Watch, PVCs will become more commonly identified and clinically evaluated.

Although previously considered mostly benign, there is growing evidence suggesting that a high PVC burden is associated with adverse cardiovascular outcomes.^1^ For instance, frequent PVCs have been associated with the development of PVC-induced cardiomyopathy, characterized by left ventricular systolic dysfunction that may improve after arrhythmia suppression,^4^, ^5^, ^6^ suggesting that a causal link may exist. Furthermore, studies have shown an association between a higher frequency of PVCs and increased risks of incident stroke,^7^ heart failure, and mortality,^8^ underscoring their clinical significance.

Over the past decade, catheter ablation has emerged as an increasingly effective therapy for patients with symptomatic PVCs or PVCs associated with structural heart disease, and those in whom medical therapy failed or is not tolerated.^1^ Advances in mapping technology, ablation tools, and operator expertise have improved both the safety and efficacy of the procedure,^1^^,^^6^^,^^9^, ^10^, ^11^, ^12^ resulting in growing adoption of ablation as a therapeutic strategy.^1^^,^^13^^,^^14^

Despite the increasing recognition of the clinical relevance of PVCs and advances in their evaluation and management, there is a striking absence of large-scale data on acute health care utilization in patients with PVCs. This knowledge gap limits our ability to understand the broader epidemiologic and economic impact of PVCs. Unlike many other arrhythmias, such as atrial fibrillation and ventricular fibrillation, where the identification has similar implications regardless of the health care setting, PVCs are so commonly observed that determining how often they rise to the level of a medical diagnosis in acute care settings is somewhat uniquely important. The current study aimed to characterize contemporary trends, and clinical and demographic characteristics of patients presenting with PVCs to acute health care facilities, and the frequency of PVC patients undergoing catheter ablation.

## Methods

### Data source, study design, and population

We used the California’s Department of Health Care Access and Information (HCAI) databases to identify all individuals aged ≥18 years who received care in a California ambulatory surgery unit, emergency department, or inpatient hospital between January 1, 2016, and December 31, 2022. We did not include years prior to 2016 because the International Classification of Diseases, Ninth Revision (ICD-9) used prior to 2016 did not have a code specific for PVCs. Each patient was assigned a record linkage number, a unique 9-digit alphanumeric value that is the encrypted form of a patient’s Social Security Number, and which was used to capture all repeated health care encounters (including across all databases) over time throughout the study period. Health care encounters with missing data regarding age, sex, and race and ethnicity were excluded.

### Study variables and diagnosis ascertainment

We gathered information on the health care setting (ambulatory surgery, emergency department, or inpatient hospital), patients’ demographics including age, sex, and race and ethnicity, and comorbidities. We used the normalized race group for a patient based on a combination of their reported race and ethnicity. If a patient’s ethnicity was “Hispanic or Latino,” then the normalized race group was coded as Hispanic or Latino. Ethnicity information from all encounters between 2005 and 2022 was combined. If Hispanic or Latino ethnicity was reported in any encounter, the patient was assigned as Hispanic or Latino. Patients with unknown, invalid, and missing ethnicity were coded as Non-Hispanic or Non-Latino. For others, the normalized race group was assigned the same value as the reported race. Hence, race was mutually exclusive and categorized as Asian/Pacific Islander, black, Hispanic or Latino, Native American (American Indian/Alaska Native), white, and “Other.” Unknown or missing race and multi-race were reclassified as “Other.” We excluded health care encounters with missing sex or an advanced age considered implausible (>120 years). Income level was estimated using the median household income for the patient’s ZIP code. Insurance status was categorized as Medicare, Medicaid, private insurance, self-pay, and others. Comorbidities of interest included hypertension, diabetes mellitus, heart failure, dyslipidemia, coronary artery disease, peripheral artery disease, ischemic stroke, chronic kidney disease, and non-PVC ventricular arrhythmias (a combination of ventricular tachycardia, ventricular fibrillation, and cardiac arrest). Diagnoses were identified through the International Classification of Diseases, Tenth Revision, Clinical Modification (ICD-10-CM) codes (Supplemental Table 1). We included both primary and secondary diagnoses. Up to 25 ICD-10-CM codes were recorded for each health care encounter. Comorbidities were assessed and updated at each health care encounter for every patient, and once they were present, they were carried forward over time. For instance, once a diagnosis of diabetes was made, the participant was considered as having diabetes at all subsequent health care encounters. PVC diagnoses were considered at each health care encounter, independent of whether it was coded in previous encounters.

Catheter ablation procedures were identified using ICD-10-Procedure Coding System and Current Procedural Terminology codes (Supplemental Table 2). A catheter ablation of PVCs was defined as a catheter ablation associated with a primary diagnosis of PVCs during the same health care encounter.

### Statistical analysis

Categorical variables are expressed as frequencies and percentages with their 95% confidence intervals , whereas continuous variables are expressed as mean with standard deviation. We assessed differences between groups using the Student’s *t* test for continuous variables and the Pearson χ2 test for categorical variables. A *P*-value of <.05 was considered significant. All analyses were performed using Stata 18 statistical package (Statacorp, College Station, TX).

### Ethical considerations

The research reported in this article was conducted in accordance with the Declaration of Helsinki and was approved by the Institutional Review Board of the University of California, San Francisco. As the study used de-identified health care data, informed consent was not required.

## Results

### Population characteristics

There were 20,280,466 people aged ≥18 years seen in an ambulatory surgery unit, emergency department, or inpatient hospital in California between January 1, 2016, and December 31, 2022. Of the 87,819,413 health care encounters recorded during this period, 235,848 (0.27%) were associated with a primary or secondary diagnosis of PVCs, whereas 22,806 (0.03%) encounters were primarily for PVCs. Patients with at least 1 health care visit with a diagnosis of PVCs were older, more likely to be male and white, and had more cardiovascular comorbidities compared with patients without a recorded diagnosis of PVCs (Table 1).

**Table 1:** Comparison of patients with and without PVCs

Variables	Total (n = 20,280,466)	Without PVCs (n = 20,081,240)	With PVCs (n = 199,226)	*P*-value
Mean age (years)	50.7 ± 20.0	50.5 ± 20.0	69.3 ± 16.2	<.0001
Female sex	11,100,028 (54.7)	11,014,010 (56.8)	86,018 (43.2)	<.0001
Race				<.0001
Asian	1,969,102 (9.7)	1,954,249 (9.7)	14,853 (7.5)	
Black	1,272,242 (6.3)	1,257,895 (6.3)	14,347 (7.2)	
Hispanic or Latino	7,785,539 (38.4)	7,734,602 (38.5)	50,937 (25.6)	
Native American	391,042 (1.9)	388,663 (1.9)	2379 (1.2)	
White	7,780,306 (38.4)	7,668,742 (38.2)	111,564 (56.0)	
Others	1,082,235 (5.3)	1,077,089 (5.4)	5146 (2.6)	
Insurance				<.0001
Medicare	3,576,903 (17.6)	3,479,496 (17.3)	97,407 (48.9)	
Medicaid	4,619,414 (22.8)	4,597,716 (22,9)	21,698 (10.9)	
Private	10,435,620 (51.5)	10,361,534 (51.6)	74,086 (37.2)	
Self-pay	971,695 (4.8)	969,458 (4.8)	2237 (1.1)	
Others	676,834 (3.3)	673,036 (3.4)	3798 (1.9)	
Income (US dollar)	65,056 ± 25,623	65,029 ± 25,611	67,827 ± 26,629	<.0001
Comorbidities				
Diabetes	3,259,736 (16.1)	3,187,521 (15.9)	72,215 (36.3)	<.0001
Hypertension	7,200,014 (35.5)	7,039,480 (35.1)	160,534 (80.6)	<.0001
Heart failure	1,415,245 (6.9)	1,332,080 (6.6)	83,165 (41.7)	<.0001
Dyslipidemia	5,024,809 (24.8)	4,893,515 (24.4)	131,294 (65.9)	<.0001
CKD	1,659,138 (8.2)	1,592,440 (7.9)	66,698 (33.5)	<.0001
CAD	1,899,305 (9.4)	1,803,565 (9.0)	95,740 (48.1)	<.0001
PAD	1,046,106 (5.2)	1,002,869 (5.0)	43,237 (21.7)	<.0001
Ischemic stroke	570,368 (2.8)	548,230 (2.7)	22,138 (11.1)	<.0001
VA	419,235 (2.1)	379,068 (1.9)	40,167 (20.2)	<.0001

Data presented are from the patient’s last recorded visit.

Categorical variables are reported as n (%).

CAD = coronary artery disease; CKD = chronic kidney disease; PAD = peripheral artery disease; PVC = premature ventricular complexe; VA = ventricular arrhythmias (ventricular tachycardia, ventricular fibrillation, and cardiac arrest).

### Temporal trends in the proportion of health care encounters with a diagnosis of PVCs

**Figure 1:**
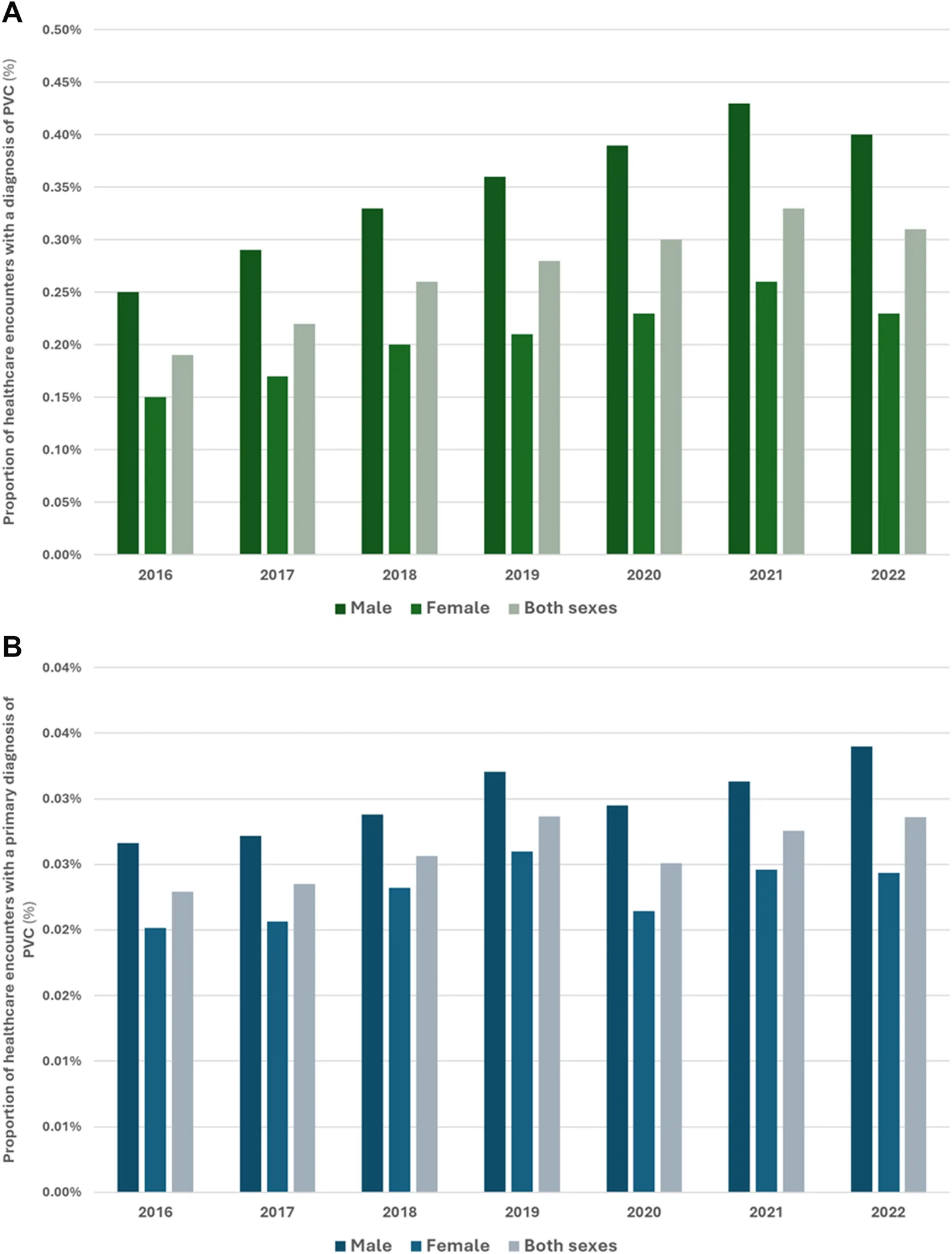
Trends in the proportion of health care encounters with a diagnosis of PVC by sex. **A:** primary and secondary diagnosis of PVC. **B:** only primary diagnosis of PVC. PVC = premature ventricular complex.

**Figure 2:**
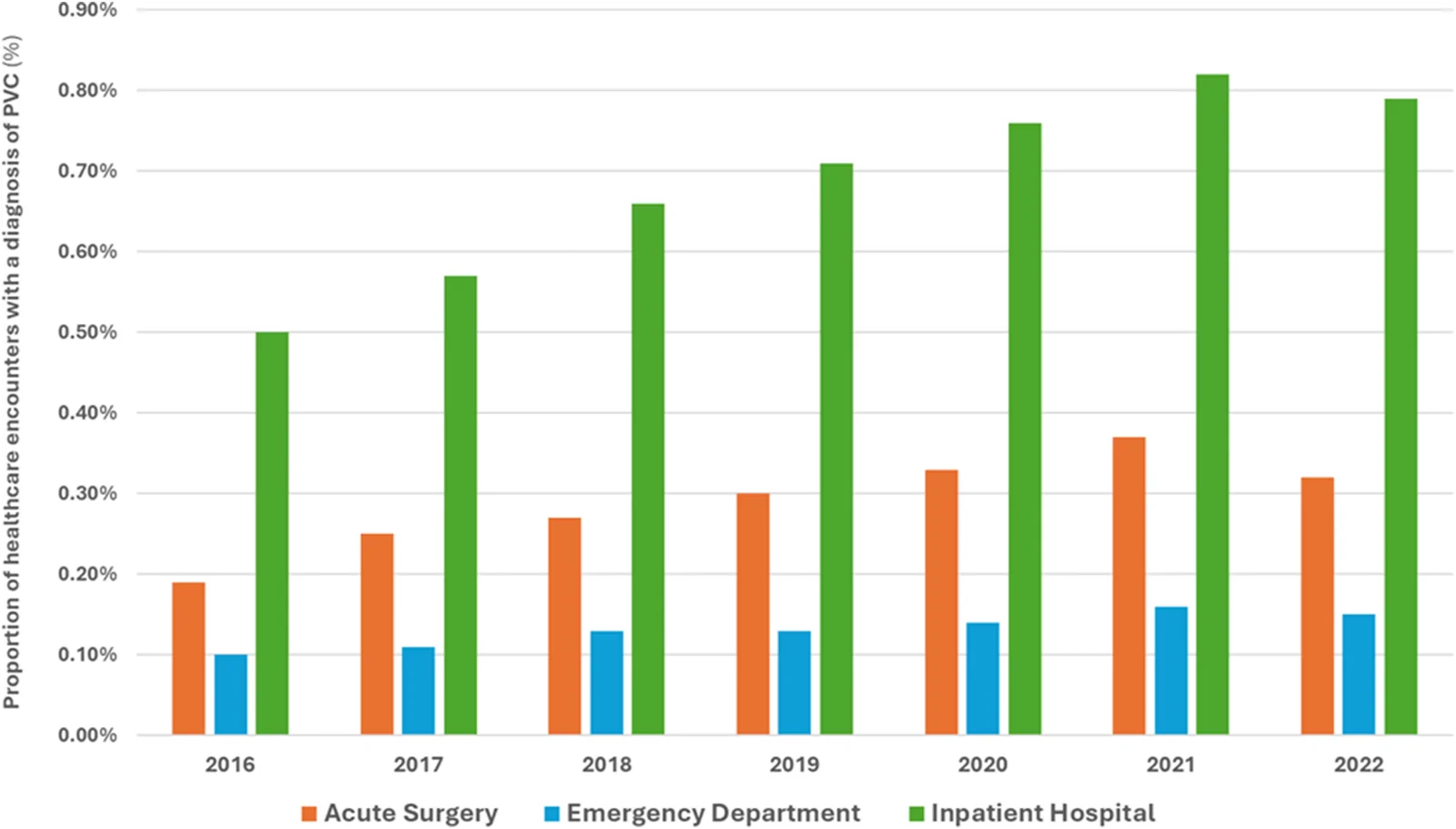
Trends in the proportion of health care encounters with a diagnosis of PVC by setting. PVC = premature ventricular complex.

**Figure 3:**
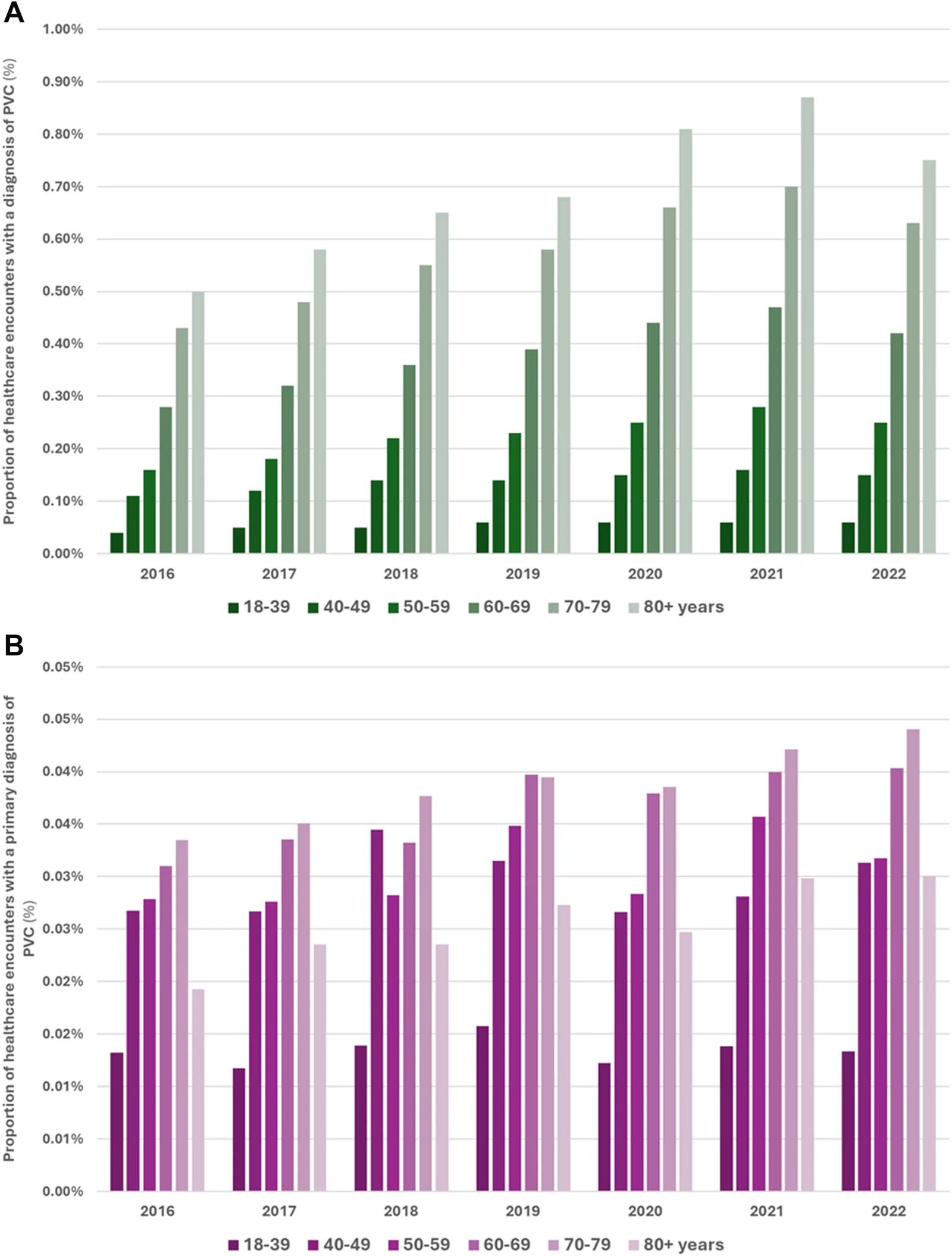
Trends in the proportion of health care encounters with a diagnosis of PVC by age groups. **A:** primary and secondary diagnosis of PVC. **B:** only primary diagnosis of PVC. PVC = premature ventricular complex.

**Figure 4:**
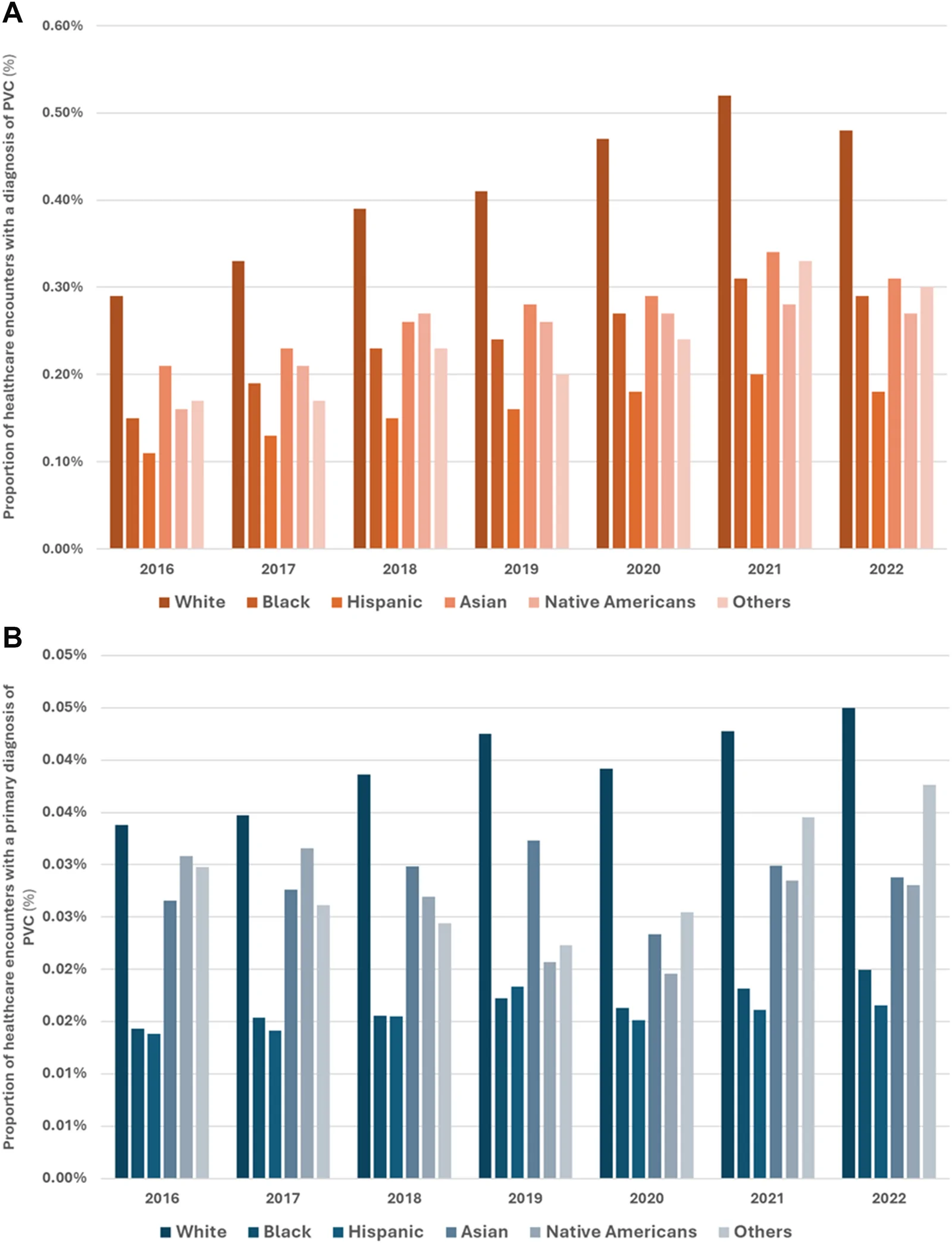
Trends in the proportion of health care encounters with a diagnosis of PVC by race. **A:** primary and secondary diagnosis of PVC. **B:** only primary diagnosis of PVC. PVC = premature ventricular complex.

**Figure 5:**
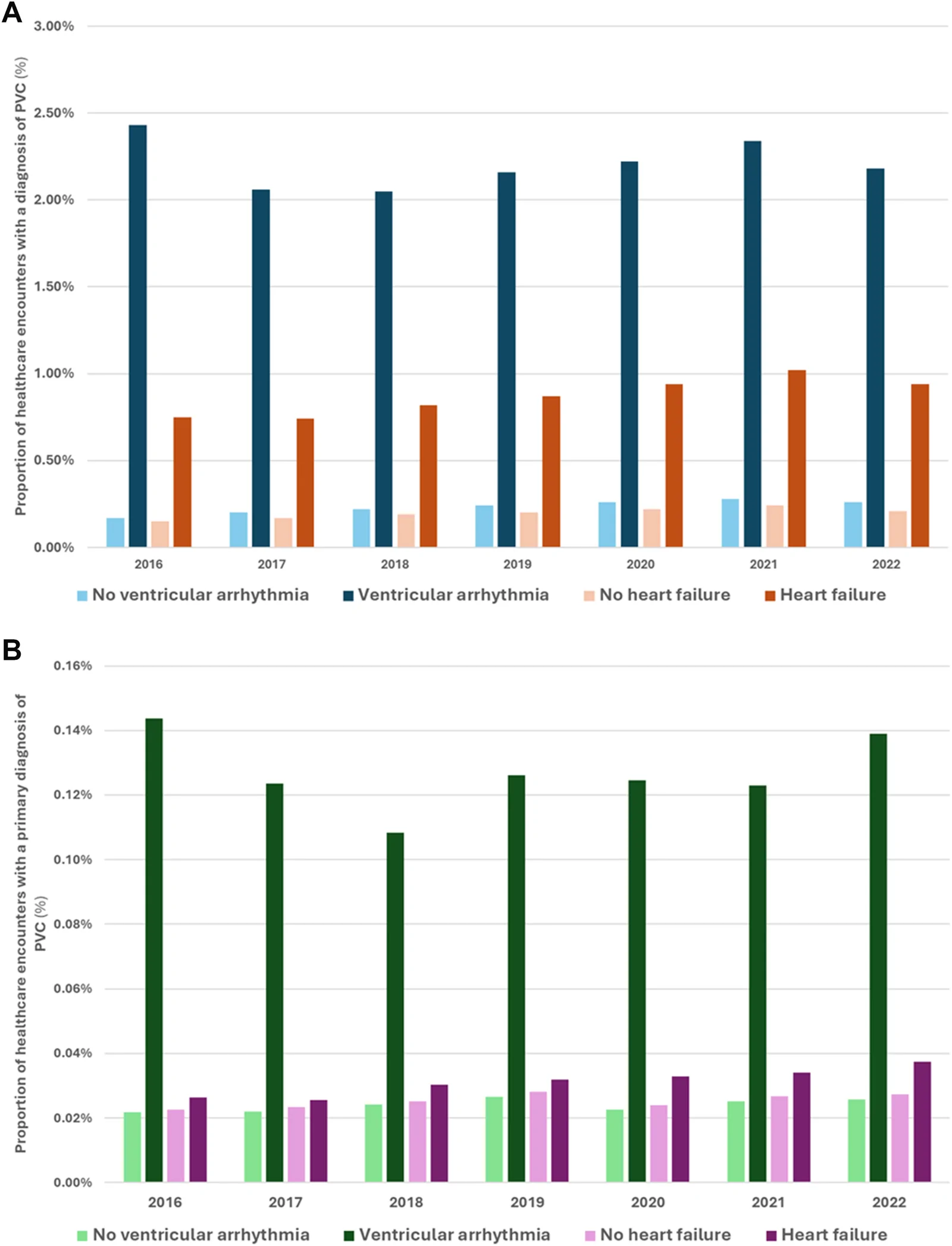
Trends in the proportion of health care encounters with a diagnosis of PVC according to history of ventricular arrhythmia or heart failure. **A:** primary and secondary diagnosis of PVC**. B:** only primary diagnosis of PVC. PVC = premature ventricular complex.

The proportion of health care encounters with primary or secondary diagnosis of PVCs increased from 0.19% in 2016 to 0.33% in 2021 and then decreased to 0.31% in 2022 (Figure 1A). This proportion was higher in the inpatient setting compared with the emergency department and ambulatory surgery unit and consistently increased over time (Figure 2). This proportion was higher in men (Figure 1A), in older individuals (Figure 3A), in white patients (Figure 4A) and increased from 2016 to 2022 in all sociodemographic groups (all *P* < .001). The proportion of health care encounters with a primary or secondary diagnosis of PVCs was significantly larger in patients with heart failure compared with those without heart failure and increased over time from 0.75% in 2016 to 0.94% in 2022 (*P* < .001). Although the proportion of health care encounters with primary or secondary diagnosis of PVCs was larger in patients with history of ventricular arrhythmias compared with those without ventricular arrhythmias (Figure 5A), this proportion decreased over time, from 2.43% in 2016 to 2.18% in 2022 (*P* < .001).

### Temporal trends in health care utilization for PVCs

The proportion of health care encounters specifically for PVCs (where PVCs were the primary diagnosis) increased over time, from 0.023% in 2016 to 0.029% in 2022 (*P* < .001, Figure 1B). This proportion was higher in men (Figure 1B), in older individuals (Figure 3B), in white patients (Figure 4B) and increased from 2016 to 2022 in all sociodemographic groups (all *P* < .001). The proportion of health care encounters for PVCs as a primary diagnosis was significantly larger in patients with heart failure compared with those without heart failure and increased over time from 0.026% in 2016 to 0.037% in 2022 (*P* < .001). This proportion was also larger in patients with history of ventricular arrhythmias compared with those without ventricular arrhythmias (Figure 5B), but decreased over time, from 0.144% in 2016 to 0.139% in 2022 (*P* < .04).

### Catheter ablation for PVCs

**Figure 6:**
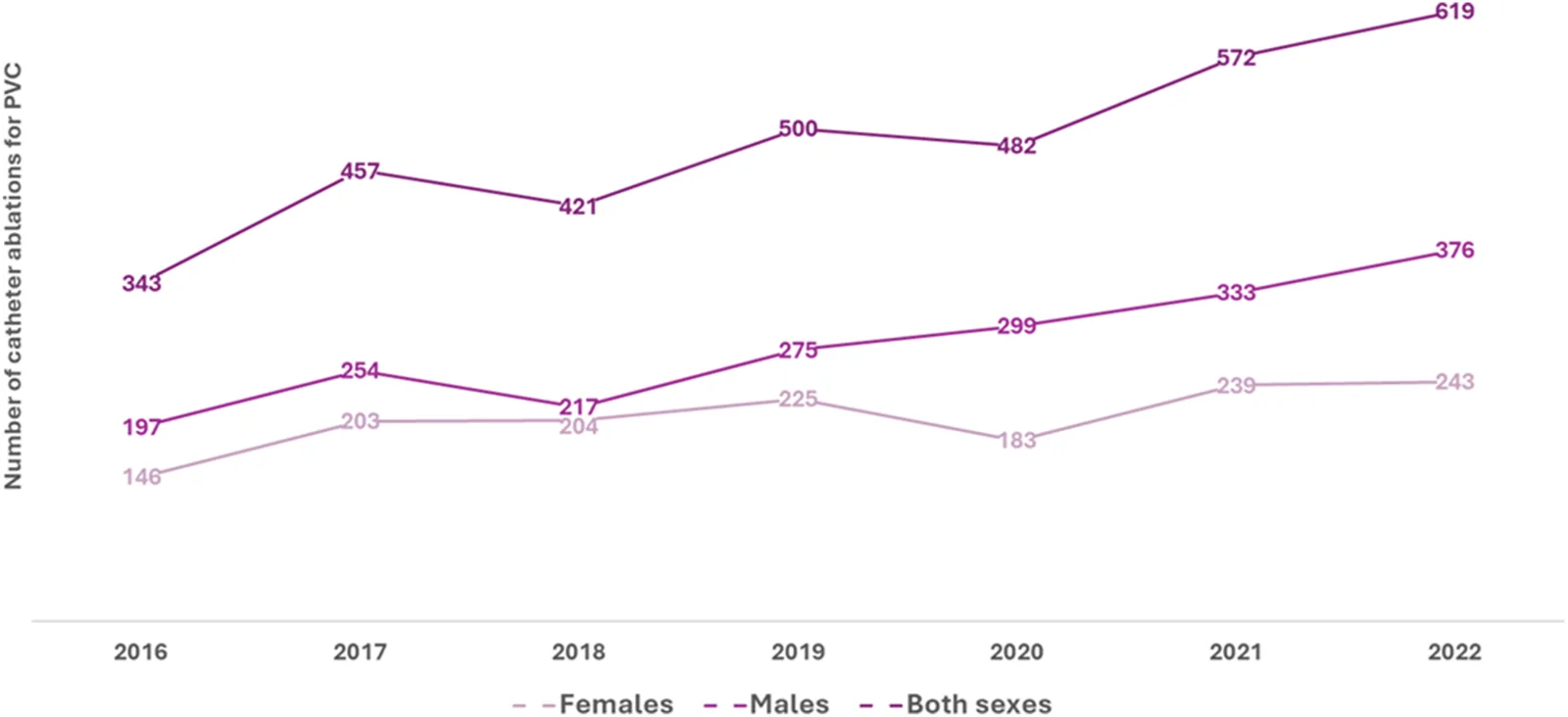
Trends in the number of catheter ablations for PVC according to sex. PVC = premature ventricular complex.

Between 2016 and 2022, 3394 catheter ablations (including 116 repeated procedures) were performed for PVCs. Of these, 1951 (57.5%) were done in male patients. The number of catheter ablations for PVCs nearly doubled from 343 in 2016 to 619 in 2022, consistently increasing in men and women (Figure 6). Of the 21,265 patients who had at least 1 health care encounter for PVCs (principal diagnosis) between 2016 and 2022, 3278 (15.4%) underwent catheter ablation for PVCs. Those who underwent catheter ablation for PVCs were older, more likely to be male and white, and had more cardiovascular comorbidities compared with who did not undergo catheter ablation (Table 2).

**Table 2:** Comparison of patients with a primary diagnosis of PVCs who underwent catheter ablation or not

Variables	Total (n = 21,265)	No catheter ablation (n = 17,987)	Catheter ablation (n = 3278)	*P*-value
Mean age (years)	59.4 ± 17.3	58.9 ± 17.7	62.1 ± 15.1	<.0001
Female sex	10,532 (49.5)	9140 (50.8)	1392 (42.5)	<.0001
Race				<.0001
Asian	1652 (7.8)	1342 (7.5)	310 (9.5)	
Black	1167 (5.5)	1054 (5.9)	113 (3.5)	
Hispanic or Latino	5532 (26.0)	4786 (26.6)	746 (22.8)	
Native American	265 (1.3)	216 (1.2)	49 (1.5)	
White	12,006 (56.5)	10,055 (55.9)	1951 (59.5)	
Others	643 (3.0)	534 (3.0)	109 (3.3)	
Insurance				<.0001
Medicare	5733 (27.0)	4608 (25.6)	1125 (34.3)	
Medicaid	2659 (12.5)	2363 (13,1)	296 (9.0)	
Private	11,973 (56.3)	10,200 (56.7)	1773 (54.1)	
Self-pay	437 (2.1)	406 (2.3)	31 (1.0)	
Others	463 (2.2)	410 (2.3)	53 (1.6)	
Income (US dollar)	70,204 ± 26,869	69,808 ± 26,881	72,381 ± 26,702	<.0001
Comorbidities				
Diabetes	4456 (21.0)	3636 (20.2)	820 (25.0)	<.0001
Hypertension	12,589 (59.2)	10,363 (57.6)	2226 (67.9)	<.0001
Heart failure	3924 (18.5)	2777 (15.4)	1147 (35.0)	<.0001
Dyslipidemia	9667 (45.5)	7,86 (43.3)	1881 (57.4)	<.0001
CKD	2821 (13.3)	2296 (12.8)	525 (16.0)	<.0001
CAD	5380 (25.3)	4076 (22.7)	1304 (39.8)	<.0001
PAD	1959 (9.2)	1669 (9.3)	290 (8.9)	.43 <.0001
Ischemic stroke	759 (3.6)	643 (3.6)	116 (3.5)	0.92
VA	2658 (12.5)	1468 (8.2)	1190 (36.3)	<.0001

Data presented are from the patient’s last recorded visit.

Categorical variables are reported as n (%).

CAD = coronary artery disease; CKD = chronic kidney disease; PAD = peripheral artery disease; PVCs = premature ventricular complexes; VA = ventricular arrhythmias (ventricular tachycardia, ventricular fibrillation, and cardiac arrest).

## Discussion

In this large, statewide analysis of administrative databases in California, we observed that PVCs were documented in approximately 0.3% of the 88 million health care encounters, and ∼1% of the 20 million patients who accessed acute care in California between 2016 and 2022. PVCs were the primary diagnosis in 0.03% of these health care encounters. Patients with a diagnosis of PVCs were significantly older, more likely to male, white, and to have cardiovascular risk factors and disease. The proportions of health care encounters with any diagnosis of PVCs, and specifically a primary diagnosis of PVCs, markedly increased over time across all demographic groups. There was also an increase in the number of catheter ablations for PVCs over time.

Our findings are aligned with previous studies that have shown an association between older age and comorbid cardiovascular conditions, especially structural heart disease, and the presence of PVCs.^1^ In the Atherosclerosis Risk in Communities (ARIC) study, older age, male sex, black race, a history of hypertension, and evidence of other heart disease were each associated with the presence of at least 1 PVC during a 2-minute ECG recording.^15^ The higher rates among white individuals in our study contrast with this prior evidence suggesting a higher risk in black patients, perhaps explained by the demographic distribution in the California population, potential disparities in health care access and cardiac monitoring, or characteristics that differ between PVCs that are detected by monitoring compared with those that result in acute care attention.

We observed a steady increase in the proportion of encounters with PVCs diagnosis from 2016 to 2021, followed by a modest decline in 2022. Several factors may explain this trajectory. Increasing awareness of the prognostic implications of frequent PVCs, along with broader availability of extended ambulatory monitoring and wearable devices,^16^ have likely contributed to rising detection in recent years. Conversely, the decline in 2022 may reflect changes in health care utilization during the COVID-19 pandemic, when non-urgent and elective visits were reduced across many health systems.^17^^,^^18^ Importantly, these trends were consistent across age, sex, and racial/ethnic groups, suggesting broad, population-wide shifts in recognition and documentation of PVCs.

PVC diagnoses were disproportionately more common in the inpatient setting compared with ambulatory surgery units or emergency departments. This finding is consistent with the higher comorbidity burden of hospitalized patients and the greater use of continuous ECG monitoring,^19^ which likely enhances PVC detection. The marked increase in PVC diagnoses among patients with heart failure is also clinically significant, supporting prior evidence that high PVC burden may worsen ventricular function and contribute to PVC-induced cardiomyopathy.^4^, ^5^, ^6^ In contrast, we observed a slight but significant decline in PVC diagnoses among patients with prior ventricular arrhythmias, which may reflect evolving management strategies, including earlier referral for ablation or improved medical therapies.

Although relatively uncommon, encounters coded with PVCs as the primary diagnosis increased significantly over the study period, from 0.023% in 2016 to 0.029% in 2022. This increase across all sociodemographic groups may reflect greater recognition of symptomatic PVCs as clinically meaningful, and heightened patient awareness of palpitations and arrhythmia symptoms. The disproportionately higher rates of PVC-related encounters among patients with heart failure further highlight the interaction between ventricular ectopy and adverse outcomes in structurally abnormal hearts.^1^

One of the most striking findings was the nearly 2-fold increase in PVC ablation procedures between 2016 and 2022. Because these procedures were counted only if they were associated with PVCs as the primary diagnosis, the absolute numbers are likely under-estimates—however, this limitation should not undermine the reliability of the relative change over time that was observed. Ablation is now recognized as a safe and effective therapy for patients with symptomatic, drug-refractory, or high-burden PVCs, particularly when associated with PVC-induced cardiomyopathy.^1^ Advances in mapping and ablation technology and tools, and operator expertise have improved both the safety and efficacy of the procedure.^1^^,^^6^^,^^9^ Indeed, success of PVC ablation procedures range from approximately 80% to 95%,^1^^,^^9^ whereas complications are observed in 0%–5% of cases.^1^^,^^6^^,^^7^^,^^10^^,^^12^ Resulting complications are mostly related to vascular access, such as hematoma, pseudoaneurysm, or atrioventricular fistula (which may often resolve without intervention).^1^ Our finding that nearly 15% of patients with health care encounters primarily for PVCs underwent ablation highlights the growing role of interventional management of PVCs in routine practice.^1^^,^^13^^,^^14^ However, demographic disparities—higher utilization among older, male, and white patients—raise important questions about potential inequities in referral patterns and access to specialized electrophysiology care.

This study has several limitations. Because our analyses were limited to acute health care settings, the demographic characteristics observed among patients with PVCs may not reflect those with PVCs in the general population. Indeed, the current study was not intended to describe a population-level assessment of PVCs, but rather acute health care utilization related to a PVC diagnosis. Our analysis included ambulatory surgery units, emergency departments, or inpatient hospitals, but not outpatient clinics. We acknowledge that some clinically relevant PVCs that were diagnosed purely in ambulatory clinics and not seen (or coded) in any of the acute health care settings included in the current databases may yet be important, and therefore the current report is, if anything, an underestimate of the true total number of encounters for PVCs. Furthermore, administrative data does not capture PVC burden, symptom severity, detailed clinical information (such as imaging, electrocardiographic monitoring, or medications) or clinical decision-making, limiting our ability to distinguish between incidental and clinically significant PVCs and fully assess the appropriateness of care. Our reliance on administrative databases using diagnosis codes may lead to ascertainment bias from coding errors, disease misclassification, and variability across institutions. However, prior studies have shown that similar use of ICD coding has a high degree of accuracy for identifying various conditions such atrial fibrillation or ventricular arrhythmias,^20^, ^21^, ^22^ and have frequently been used in high-impact and well-validated epidemiologic studies.^23^^,^^24^ In addition, the absence of procedure-specific codes distinguishing PVC from ventricular tachycardia ablation may result in misclassification and potential underestimation of PVC ablation procedures, particularly when PVCs are not coded as the primary diagnosis. The observed temporal trends may have been influenced by changes in coding practices, with underreporting in the early years of the study, especially with the transition from ICD-9 to ICD-10 codes in 2015. These trends may also reflect increased detection of PVCs because of the expanded use of cardiac monitoring technologies and greater clinician awareness of their clinical significance. Although the current databases ostensibly capture essentially every catheter ablation in California, the databases unfortunately do not include information on procedural indications, medications, or the relative efficacy of various therapies.

## Conclusion

This statewide analysis demonstrates that PVCs, though infrequently coded as a primary diagnosis, represent an important and increasing component of health care utilization in California. The burden is particularly high among older adults and those with cardiovascular comorbidities, especially heart failure. The rising number of ablation procedures highlights the expanding role of interventional management, whereas demographic disparities suggest potential gaps in access to specialized care. These findings provide contemporary insights into PVC-related care and highlight the need for further research into optimal strategies for diagnosis, risk stratification, and treatment of PVCs. These findings also suggest that effective prevention strategies might meaningfully minimize resultant acute health care utilization.

## Disclosures

Dr Marcus reports receiving funding from the NIH, PCORI, and the California Department of Cannabis Control; he is a consultant for and equity holder in InCarda.

## Footnotes

Appendix Supplementary data associated with this article can be found in the online version at https://doi.org/10.1016/j.hroo.2026.04.026.
