# Supplementary material for: Health care utilization for premature ventricular complexes in acute settings
Source: Heart Rhythm O2. 2026 May 8;7(8):1518–27. doi: 10.1016/j.hroo.2026.04.026 (PMC13523593; doi:10.1016/j.hroo.2026.04.026)
Supplement: Supplementary file 1 [file mmc1.docx]

**APPENDIX**

**Healthcare Utilization for Premature Ventricular Complexes in Acute Settings**

**Table S1**. International Classification of Diseases, Tenth Revision, Clinical Modification (ICD-10-CM) codes

| **Diagnoses** | **ICD-10** |
| --- | --- |
| Cardiac arrest | I46 |
| Chronic kidney disease | I12, I13, N18 |
| Coronary heart disease | I20-I25, Z95.1, Z95.5, Z98.61 |
| Diabetes mellitus | E10-E14 |
| Dyslipidemia | E78 |
| Heart Failure | I11.0, I13.0, I13.2, I50 |
| Hypertension | I10-I16, I67.4 |
| Ischemic stroke | I63, G46 |
| Peripheral artery disease | I70, I73.9 |
| Premature ventricular complexes | I49.3 |
| Ventricular tachycardia | I47.2 |
| Ventricular fibrillation | I49.01 |

**Table S2**. International Classification of Diseases, Tenth Revision, Procedure Coding System (ICD-10-PCS) and Current Procedural Terminology (CPT) codes for catheter ablation of premature ventricular complexes

| **ICD-10-PCS** | **CPT** |
| --- | --- |
| 02583ZZ, 02584ZZ, 02583ZF | 93654 |
